# British Columbia Hospitals: examination and assessment of payment reform (B-CHeaPR)

**DOI:** 10.1186/1472-6963-11-150

**Published:** 2011-06-24

**Authors:** Jason M Sutherland, Kimberlyn M McGrail, Michael R Law, Morris L Barer, R Trafford Crump

**Affiliations:** 1 University of British Columbia, Faculty of Medicine, Centre for Health Services and Policy Research, 201 - 2206 East Mall, Vancouver, B.C., V6T 1Z3, Canada

## Abstract

**Background:**

Accounting for 36% of public spending on health care in Canada, hospitals are a major target for cost reductions through various efficiency initiatives. Some provinces are considering payment reform as a vehicle to achieve this goal. With few exceptions, Canadian provinces have generally relied on global and line-item budgets to contain hospital costs. There is growing interest amongst policy-makers for using activity based funding (ABF) as means of creating financial incentives for hospitals to increase the 'volume' of care, reduce cost, discourage unnecessary activity, and encourage competition. British Columbia (B.C.) is the first province in Canada to implement ABF for partial reimbursement of acute hospitalization. To date, there have been no formal examinations of the effects of ABF policies in Canada.

This study proposal addresses two research questions designed to determine whether ABF policies affect health system costs, access and hospital quality. The first question examines the impact of the hospital funding policy change on internal hospital activity based on expenditures and quality. The second question examines the impact of the change on non-hospital care, including readmission rates, amount of home care provided, and physician expenditures.

**Methods/Design:**

A longitudinal study design will be used, incorporating comprehensive population-based datasets of all B.C. residents; hospital, continuing care and physician services datasets will also be used. Data will be linked across sources using anonymized linking variables. Analytic datasets will be created for the period between 2005/2006 and 2012/2013.

**Discussion:**

With Canadian hospitals unaccustomed to detailed scrutiny of what services are provided, to whom, and with what results, the move toward ABF is significant. This proposed study will provide evidence on the impacts of ABF, including changes in the type, volume, cost, and quality of services provided. Policy- and decision-makers in B.C. and elsewhere in Canada will be able to use this evidence as a basis for policy adaptations and modifications. The significance of this proposed study derives from the fact that the change in hospital funding policy has the potential to affect health system costs, residents' access to care and care quality.

## Background

Accounting for $46-billion - or 36% of public spending on health care [1] - hospitals are a major target for cost reductions through efficiency initiatives in Canada. Some provinces are considering payment reform as a vehicle to achieve this goal. The use of financial incentives to increase hospital efficiency is now widespread in Europe and has occurred in the United States (US) since 1983 [2-4]; however, the approach remains largely untested in Canada [5].

In April 2010, the British Columbia (B.C.) provincial government implemented activity based funding (ABF) for hospitals, marking a fundamental change to the method of funding acute hospital care. ABF will direct up to 20% of available funds to acute hospitals on the basis of types and volume of services. This change is significant in an industry accustomed to historically-based global budgets and unaccustomed to detailed scrutiny of what services are provided to B.C.'s 4.5 million residents and at what cost.

While this change in hospital funding is being implemented in B.C., a debate over the relative merits of hospital ABF is unfolding across Canada [6,7]. For example, the Canadian Medical Association and the B.C. Medical Association have recently staked positions supportive of ABF [8-10], although support for ABF among the medical community is mixed [11,12]. The Canadian Nurses Association supports an increasing emphasis on output measurement [13] but falls short of endorsing ABF, while other groups are calling for more comprehensive baseline data [14]. Although there has been no formal examination of the effects of ABF policies in Canada - in part because B.C. is the first province to adopt ABF for acute hospitals on a large scale - several other provinces are considering adopting similar funding models.

This project will provide an evidence base on the impacts of ABF, including changes in the type, volume, cost, and quality of services provided. Policy- and decision-makers in B.C. and elsewhere in Canada will be able to use this evidence as a basis for policy adaptations and modifications.

### Rationale

#### What is Activity Based Funding?

In B.C., regional Health Authorities (HA) are responsible for providing health services to their region's population. The HAs derive their operating revenue from multiple sources, though the vast majority is provided by the provincial government [15,5]. Provincial funds are allocated to HAs through a mix of: 1) block operating grants (which are historically- and population-based, independent of the volume and type of activity provided to the region's residents), 2) targeted line items linked to specific policy objectives, and 3) to a very minor degree, pay for performance initiatives [16]. Health authorities then allocate from their block grants to the various community-based providers (excluding physicians) in their regions, with a significant portion of those resources going to acute care hospitals.

ABF is a variant of fee-for-service in which funds are allocated based on the volume and type of services provided, including considerations of patient case-mix. ABF is an alternative to the traditional Canadian block operating grant funding for hospitals but has been widely used outside of Canada for some time now [2-4]. A small-scale implementation in Canada was abandoned by the Ontario Ministry of Health and Long-Term Care [17] in 2007 due to poor data quality [18,19] and lack of support across affected stakeholders. In the B.C. program, as an addition to a base block grant, a portion of hospital funding will flow based on the number of cases, with remuneration adjusted for the mix of patient diagnoses and the services and procedures provided to those patients.

#### Information Base for Supporting ABF

The success of ABF is critically linked to the ability to measure 'weighted' hospital output accurately. Methods for characterizing hospital output according to the patients' mix of clinical conditions and interventions have become common and are known as case mix adjustments. The most popular method is diagnosis related groups, or DRGs [20], though variants of DRG have been developed, each customized to address local policy and health delivery characteristics and objectives [21,22]. In Canada, all acute inpatient discharge data submitted to the Canadian Institute for Health Information's (CIHI) Discharge Abstract Database (DAD) are case-mix-adjusted using their Case Mix Group (CMG+) methodology [23]. Case mix adjustment is important since it weights hospital discharges according to their expected relative use of resources (and therefore cost). Each CMG+'s expected relative cost is represented by a resource intensity weight (RIW). As the length of stay has no bearing on the RIW value assigned (with minor exceptions for patients with exceptionally long lengths of stay), the incentive is to shorten lengths of stay in order to keep actual costs below the RIW-based case-specific relative amounts.

The veracity of weighting hospital output by RIW is contingent on accurately reported clinical data. CIHI's clinical chart re-abstractions have shown that Canadian hospitals have modified their clinical coding behaviour to maximize RIWs even in the absence of direct financial incentives to do so [18,19]. This behavior introduces an additional challenge to our study and requires us to be able to differentiate actual changes in activity mix as a result of the changing financial incentives from apparent changes in activity mix that are merely shifts in coding practices.

#### Gaps in knowledge

The introduction of payment tied to particular types of activity represents a major change in hospital payment policy in Canada. We lack a knowledge base in Canada regarding the actual effects of these policies; and the (also relatively sparse) lessons from other health care systems cannot be necessarily generalized to the Canadian context. The natural experiment that is unfolding in B.C. thus provides an important opportunity to examine whether the theoretical incentive effects of ABF for hospitals, tied to efficiencies sought by B.C.'s Ministry of Health Services (MoHS), will actually materialize.

This project also provides a first-ever opportunity to examine the subsequent impact of ABF on the non-hospital sector. From a policy perspective, our results will provide as close to real-time impact information to B.C. decision makers as is possible in the world of health services research regarding the effects of ABF on costs, quality and access to care for residents.

### Study Objective

The primary objective of this study is to examine the impact of ABF on acute care hospitals and related services in B.C. This objective will be fulfilled by meeting two specific aims.

**Aim 1**: To measure internal (to the hospital) changes resulting from the shift toward ABF. In a longitudinal analysis of observational data, we will measure whether and how hospitals respond to the changed financial incentives. Related hypotheses include:

**Hypothesis 1.1**: Lengths of stay will decrease and case mix adjusted volume of patients will increase from baseline levels. We will test for changes in trend over time.

**Hypothesis 1.2**: There will be changes in in-hospital quality measures (mortality and adverse events) for specific conditions that are characterized by their variability in clinical utilization patterns. We will test for changes in trend over time.

**Hypothesis 1.3**: Hospitals' expenditures will increase from baseline levels. We will construct a model to detect changes in trend over time of hospital expenditures.

**Aim 2**: To measure external (to the hospital) changes. In a longitudinal analysis of observational data, we will measure whether other components of the health care system are affected by the shift toward ABF. Related hypotheses include:

**Hypothesis 2.1**: There will be an increase in 30 day readmission rate, rate of admissions from emergency department (ED) and rate of admissions for ambulatory sensitive conditions, all indicators of sub-optimal acute care. We will test for an increase in the rates over time.

**Hypothesis 2.2**: There will be an increase in the number of new home care patients subsequent to being discharged from acute care. We will construct a model to detect changes in the trend of: 1) new home care patients over time and 2) readmissions to acute care from home care over time.

**Hypothesis 2.3**: There will be an increase in expenditures on physician services. We will test for an increase in physician expenditures over time.

## Methods/Design

### Design

This study has been approved by the University of British Columbia's Research Ethics Board.

#### Aim 1

We do not expect ABF to affect all patient types uniformly. Conditions for which patterns of care are well defined are expected to be invariant to hospital funding incentives. Accordingly, we will map all hospitalizations into one of three 3 care types; *supply sensitive care, preference sensitive care *and *evidence-based care*. The rationale for stratifying analyses into these 3 categories is that utilization intensity has been shown to vary by condition, region and clinician preference [24]. For each stratum, the change in the average length of stay and number of discharges is expected to vary (as shown in the 2^nd ^column of Table 1).

**Table 1:** Expected response of hospitals to ABF by type of care provided

Care Type	Expected Response	Reason for Hospitalization
**Supply Sensitive**	Some Response	Congestive heart failure
		Cancer
		Chronic lung disease
	
**Preference Sensitive**	Unknown	Benign prostatic hypertrophy
		Hip replacement
		Knee replacement
	
**Evidence-based Care**	None	Appendectomy
		Leg fracture

In addition to overall case mix adjusted volume and day surgery volume, we will calculate average lengths of stay for case mix adjusted volume of patients for each condition listed in the third column of Table 1. We will use ICD-10-CA codes underlying the CMG; a strategy for identifying hospitalizations that ensures that the conditions identified in Table 1 are the primary reason for hospitalization (and removes patients whose hospitalization is for reasons other than those listed).

In-hospital quality and outcomes as a result of a shift to ABF will be monitored in B.C. and shared with the MoHS. Based on broadly accepted in-hospital quality indicators [25], our hospital quality indicators will be mortality rates for selected medical and surgical patients (shown in Table 2), overall mortality rates plus the number of patient safety and adverse events [26].

**Table 2:** Conditions for calculating age, sex and risk adjusted in-hospital mortality rates

Type	Title	Method for Identifying Cases
**Medical**	Acute myocardial infarction	ICD-10-CA codes
	Congestive heart failure	
	Stroke	
	Gastrointestinal hemorrhage	
	Pneumonia	
	
**Surgical**	Esophageal resection	CCI codes
	Pancreatic resection	
	Carotid endarterectomy	
	Craniotomy	
	Hip replacement	

Hospital mortality rates will be calculated as the number of inpatient deaths relative to the number of separations for the conditions listed in Table 2. Methods for calculating mortality rates for surgical patients are based on the Leapfrog Group's evidence-based hospital referral (EBHR) standards for surgery, while mortality rates for medical conditions are based on low mortality patient groups [27] for which mortality is an unexpected outcome. We will adjust hospital mortality rates for differences between hospitals of age, sex and risk profiles of patients [28,29].

We will also calculate in-hospital event rates, representing unintended injuries or complications of care [30] per volume of surgical separations and analyze changes in trend over time. These rates include: *post operative sepsis (surgical site infection); post operative thrombo-embolism; *and *repeat trips to the OR*. These quality indicators are commonly applied and can be readily computed with B.C.'s hospital separation data.

Finally, we will adjust hospital expenditure data to control for factors associated with price inflation over time, such as labour contract increases, in order to isolate ABF-related changes in expenditures.

#### Aim 2

Since all segments of the health care system are intertwined, even if funding streams are not, this study provides a first-ever opportunity to develop knowledge regarding the non-hospital consequences of ABF. To examine health system effects, we will calculate three categories of indicators: *early discharge from hospital *(resulting in readmission to hospital); *access to care; *and (inappropriate) *admissions for ambulatory care sensitive conditions *(see Table 3).

**Table 3:** Indicators of early discharge, access and admissions for ambulatory care sensitive conditions

Impact	Condition	Level of Reporting
**Readmission**	All Cause	Hospital
	Acute myocardial infarction	Hospital
	Prostatectomy	Hospital
	
**Access**	Emergency department admission rate Hip fracture surgery within 2 days of admission	Hospital
	Hip fracture surgery within 2 days of admission	Hospital
	
**Ambulatory care sensitive conditions**	Dehydration: hospital admission rate	Health Authority
	Diabetes mellitus: hospital admission rate	Health Authority

Using hospital data for all three categories of indicators, we will measure the association between rates of acute hospital readmissions and ABF. We will look for evidence of a change in 30 day hospital readmission rates for all causes, acute myocardial infarction (AMI) and prostatectomy discharges. Risk adjusted AMI readmission rate has been linked to the types of drugs prescribed at discharge, post-discharge therapies and the quality of follow-up care, whereas prostatectomy readmission rates provide one measure of follow up care [31,32]. These indicators are often used to signal too-early discharge. We will use ICD-10-CA diagnosis and Canadian Classification of Health Interventions (CCI) procedure codes to identify patients.

We will calculate indicators of system level effects on access: the rate of admissions from emergency department and the risk-adjusted rate of hip fracture in patients aged 65 and older who underwent hip fracture surgery on the day of admission or the next day. Increases in hospitals' emergency department utilization are linked to (less) availability of out-of-hospital care while delayed hip fracture surgery has been associated with a lack of resources, physician unavailability and other issues related to access to care [31].

We will calculate rates of hospital admissions for two ambulatory sensitive conditions: dehydration [33] and diabetes mellitus [33]. We believe changes in these rates are important signals of care quality and potentially negative implications for quality of life [34]. We are selecting these two conditions due to their broad use plus our ability to derive these indicators from B.C.'s hospital separation data. We will use the ICD-10-CA diagnoses to identify these patients.

Previous studies have not rigorously examined the non-hospital effects of changing acute hospital payment methods, and it is not known how the change to ABF will affect the demand for home care or the ability to maintain high quality home care services. We will calculate the number of new home care patients each month using the continuing care dataset, looking retrospectively to determine which patients are 'new' to home care as opposed to existing patients. Changes in the trend of the number of new home care patients will provide valuable insight into the acuity of patients discharged from acute care. Based on the cohort of home care patients, we will then calculate the number of readmissions to acute care from home care over time. This important signal of care quality may suggest increased pressure on home care services attributable to ABF.

To date, no published information describes the nature of change in use of physician services after implementation of ABF. Medical Services Plan data allow us to determine the amount of all fee-for-service physician payments. For each acute discharge, we will link the amount of physician payments in the 30-day period following the incident discharge (including readmissions). Total physician expenditures will be linked to hospital data and registry data to enable our analytic dataset to be able to be analyzed over time and for specific conditions (see Table 3). To isolate changes of physician utilization from physician payments over time, we will draw on work currently being undertaken at the Centre for Health Services and Policy Research (CHSPR) on a separate study to remove the effects of fee changes from physician fees.

### Data Resources and Variable Construction

The analytic dataset for **Aim 1 **(*internal to the hospital*) will be derived from acute and outpatient DAD hospitalizations linked to the registry data. The dataset will include the following variables: month and year of discharge, CMG, CIHI-defined age group, hospital, region of residence (HA), length of stay (LOS), ICD-10-CA codes, indicator of death, and Adjusted Clinical Groups (a measure of health status [28]). The number of inpatient separations per year, for the conditions to be studied (Table 1), is expected to exceed 5,000. We will analyze hospital separations over the period from fiscal year 2005/2006 through to, and including, 2012/2013, to ensure consistent application of CMG+ across all years.

We will consider the effect of data quality and completeness on the results. The most recent multi-stage randomized studies of DAD data quality, which involve clinical chart re-abstractions, have been conducted by CIHI and have found the majority of data elements in the DAD are accurately reported [35], with comorbidity reporting being an important exception. We will use the results of these studies to exclude those ICD-10-CA codes susceptible to being affected by coding practice changes. Further, we will link Medical Services Plan data to hospital separation data to evaluate completeness of reporting (a means to validate procedure information in the DAD).

One comprehensive analytic dataset will be created for **Aim 2 **(*external to the hospital*) for the years 2005/2006 through 2012/2013. This analytic dataset will be the product of linking acute hospital, registry and continuing care datasets with summarized physician fee-for-service expenditure data. The hospital data will be used to calculate rates of: readmission, admissions from the emergency department and ambulatory care sensitive admissions. The analytic dataset will include admission date, CMG, hospital, region of residence, ICD-10-CA codes, procedure date and whether the patient was admitted from the ED. Readmissions at separate hospitals will be attributed back to the incident hospitalization. The analytic dataset will include an indicator variable to represent incident cases of new home care in the period immediately following discharge from acute care. A variable representing the amount of physician services will be determined from the physician fee-for-service Medical Services Plan (MSP) data linked to patient data.

We recognize that approximately 20% of physicians are not paid by on a fee-for-service basis, so we will limit our inferences to physicians and expenditures paid by fee-for-service as we expect salaried physicians to be less likely to be affected by the incentives of ABF. We will identify those physicians paid primarily by fee-for-service using an information resource being developed at CHSPR for a separate project.

### Analysis

Initially, we will provide descriptive analyses of the overall trend in provincial LOS and case mix adjusted volume. We will then construct a multivariate zero-truncated Poisson regression model to identify factors associated with length of stay for each condition included in Table 1. The dependent variable, length of stay of patient *i*, is *Y*_*i*_. The regression model effects include: hospital and patient level effects, ***X***_***i***_, (such as age and ACG). Hospital level clustering will be incorporated into the model. The model is:

where time and policy (and interaction between time and policy) represent study month (1,2,3...) and an indicator variable for ABF, respectively.

If the MoHS changes ABF policy during the study period (e.g., to 30% of acute funding), we will change the parameterization of the model to reflect the change. The parameters of interest are θ and τ, where θ represents an immediate change in the length of stay and τ represents a change in the trend in length of stay. The results will be presented as absolute and relative changes to facilitate interpretation. While we are confident these methods are appropriate for the data and the questions being addressed, if small sample sizes inhibit analysis, we will explore composite measures (which combine multiple conditions) for standardized rates [36-38].

We will use a linear regression model to examine the relationship between ABF and hospitals' case mix adjusted volume of cases (transformed for normality if needed). The dependent variable, *Y*_*i*_, is the hospital's monthly number of case mix adjusted cases. Repeated measures (clustering) on hospitals over time will be incorporated into the model through ***X***_***i***_, a vector of hospital effects. The regression equation is written:

where the parameters of interest are change in level (θ) and change in trend (τ). We will construct a regression model for overall weighted cases plus a separate regression model for each condition included in Table 1.

We will model the number of in-hospital medical and surgical deaths, *Y*_*i*_, (as described in Table 2) relative to the total number of separations, *N*_*i*_, using a Poisson model for count data. The vector ***X***_***i ***_will include hospital effects and adjust for repeated measurements on the same hospital. The regression model is written:

The parameters of interest are those associated with immediate change in the number of deaths (relative to the number of discharges, *N*_*i*_) and a change in the trend of number of deaths. We will explore the use of zero-inflated Poisson (ZIP) models if we find an abundance of counts of 0.

Similar to the approaches described above, we will use a linear regression model (suitably transformed for normality) to detect changes in hospital expenditures associated with ABF implementation (including adjustments for effects of inflation) and controlling for repeated measures on hospitals.

Following published methods for calculating hospital readmission rates [31], we will calculate overall readmission rates using a multivariate Poisson model for count data, controlling for repeated measures on hospitals. We will also construct separate models for the conditions listed in Table 3. The regression models of monthly hospital readmission rates will include effects for hospital, time and a binary variable for ABF. The parameters of interest represent immediate change in readmission and change in trend over time.

We will use a linear regression model to examine the relationship between the number of (monthly) new home care patients and ABF. The unit of observation will be the number of new home care patients within an HA and we will control for repeated measures over time. The effects of primary interest are those associated with immediate change in new home care patients and a change in the trend of home care patients. Given stable monthly count data at the HA-level, we expect to be powered able to detect moderate and large sized effects.

Similar to the methods described above, we will model the number of monthly readmissions to acute care from home care using a Poisson model for count data and adjust for HA and hospital effects. The parameters of interest are those associated with immediate change in the number of readmissions to acute care from home care and a change in the trend of number of readmissions.

We will also use a log-transformed model to examine the association between patients' fee-for-service physician expenditures (fee-adjusted) over time, and implementation of ABF. The regression equation for patients' fee-for-service, fee-adjusted, physician expenditures is written:

where the parameters of interest are change in level (θ) and change in trend (τ). The vector ***X***_***i ***_will include effects to control for repeated measures on physicians and hospitals plus will adjust for patient level effects, such as age and comorbidity burden (derived from CMG).

## Discussion

The examination of hospital and non-hospital effects of the implementation of ABF in B.C. is both critical and timely given the high public profile of the "sustainability" of the health care system, the large cost footprint represented by acute care hospitals, and the mushrooming interest in ABF across Canada. For the MoHS, the policy rationale for ABF - to increase hospital efficiency while holding the line on aggregate expenditures - is clear: regional HA block operating grants fail to create the incentives for hospital-derived efficiencies that ABF may provide.

If B.C.'s policy-makers are to assess the extent to which their objective of increasing hospital efficiencies without sacrificing quality has been achieved, it is critical that they be able to monitor effects of ABF on a timely basis. While from a policy perspective, this proposed work will help position the MoHS to ensure its objectives are met, the overall goal of this research is to examine the impact of ABF not just on hospital activity, but more broadly on the health care system overall.

### Study Limitations

While we are leveraging the natural experiment of a significant change in hospital funding, we are limited in the type of study design we can use. Since the MoHS' ABF initiative includes all hospitals, it is not possible to 'randomize' hospitals to ABF and non-ABF groups as to isolate the effect of the implementation of ABF. We are not including another province's hospitals to act as a 'control' group due to the potential confounding from reporting differences between provinces, hospital funding policy changes in other provinces and the potentially insurmountable problems associated with, but undoubtedly lengthy time required to, securing data access in multiple provinces. Given these constraints, a longitudinal design is the strongest possible design. Our study will be limited to administrative datasets, precluding the use of detailed clinical data that might inform our analyses.

### Potential Contributions and Significance

This project represents a unique opportunity to examine the health care system during a time of significant policy change in B.C. that will have implications for health system funding across Canada. This project will lay a solid foundation upon which to build future projects, including the development and integration of outcome and quality indicators into health system performance measurement in B.C.

This study represents a unique opportunity to leverage the natural policy experiment occurring in B.C. and will contribute to understanding the dynamics underlying the most significant and expensive segments of our health care system. By the end of the study, we expect to propose a series of policy recommendations as they relate to monitoring health system use within a new framework for hospital funding. The insight gained from these activities will be of high value to all Canadians.

## Competing interests

The authors declare that they have no competing interests.

## Authors' contributions

JS is responsible for the intellectual content of the study proposal; he conceived, developed, and articulated the conceptual framework, study design and statistical analysis of data. JS was the primary author of the funded study proposal and gave his final approval of the version to be submitted. KM made substantial contributions to the study design and study proposal drafts. ML made substantial contributions to the study design and study proposal drafts. MB made substantial contributions to the study design, study proposal drafts and gave his final approval of the version to be submitted. TC compiled and edited the study proposal for publication. All authors have read approved the final manuscript.

## Pre-publication history

The pre-publication history for this paper can be accessed here:

http://www.biomedcentral.com/1472-6963/11/150/prepub
